# Integrative Korean medicine treatment for low back pain with radiculopathy caused by Bertolotti syndrome: A CARE-compliant article and retrospective review of medical records

**DOI:** 10.1097/MD.0000000000039720

**Published:** 2024-09-13

**Authors:** Sohyun Cho, Yong-Jun Ahn, Yoon Jae Lee, In-Hyuk Ha, Ye-Seul Lee

**Affiliations:** a Jaseng Korean Medicine Hospital, Seoul, Korea; b Jaseng Spine and Joint Research Institute, Jaseng Medical Foundation, Seoul, Korea.

**Keywords:** Bertolotti syndrome, case report, complementary alternative medicine, Korean medicine, lumbosacral transitional vertebrae

## Abstract

**Rationale:**

Bertolotti syndrome (BS) is characterized by radiculopathy caused by structural anomalies. Despite the structural deformity, conservative treatment is predominantly recommended due to surgery-related complications. Because of the diagnosis complexity, the incidence and contributing factors of BS, remain controversial. We report the case of a patient with BS who was treated with integrative Korean medicine (IKM). Moreover, we evaluated the epidemiological characteristics of lumbosacral transitional vertebrae (LSTV) from medical records of patients diagnosed with LSTV at 4 different medical clinics of Korean medicine.

**Patient concerns:**

A 33-year-old male patient with low back pain and severe radiculopathy was diagnosed with BS (Castellvi Type II) on magnetic resonance imaging at a local orthopedic clinic. Additionally, the medical records of patients with BS who had been treated with IKM in 4 different institutions of Korean medicine were analyzed, and the characteristics of patients suffering from BS were identified.

**Diagnoses, interventions, and outcomes:**

The patient underwent IKM treatment for 40 days as an inpatient. The patient’s condition was assessed using the Euroqol 5-dimension index and Oswestry Disability Index, and symptom severity was measured using the Numeric Rating Scale. IKM was effective in improving pain and functional disability without causing any adverse effects. In a retrospective review of medical records, the study identified symptom trends reported by patients with LSTV.

**Lessons:**

IKM demonstrates potential efficacy in BS management, with notable trends in LSTV-related symptomatology warranting further investigation.

## 1. Introduction

**Figure 1.**
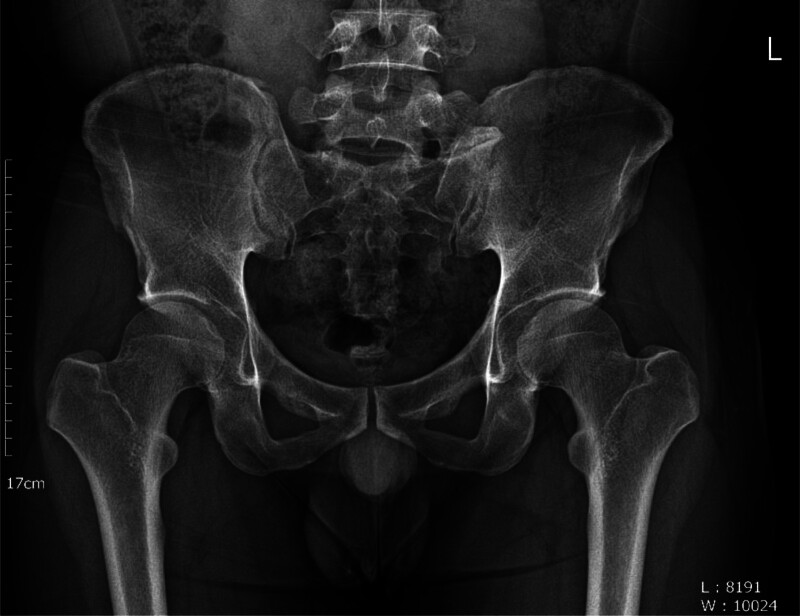
Radiographic findings depicting Bertolotti syndrome.

**Figure 2.**
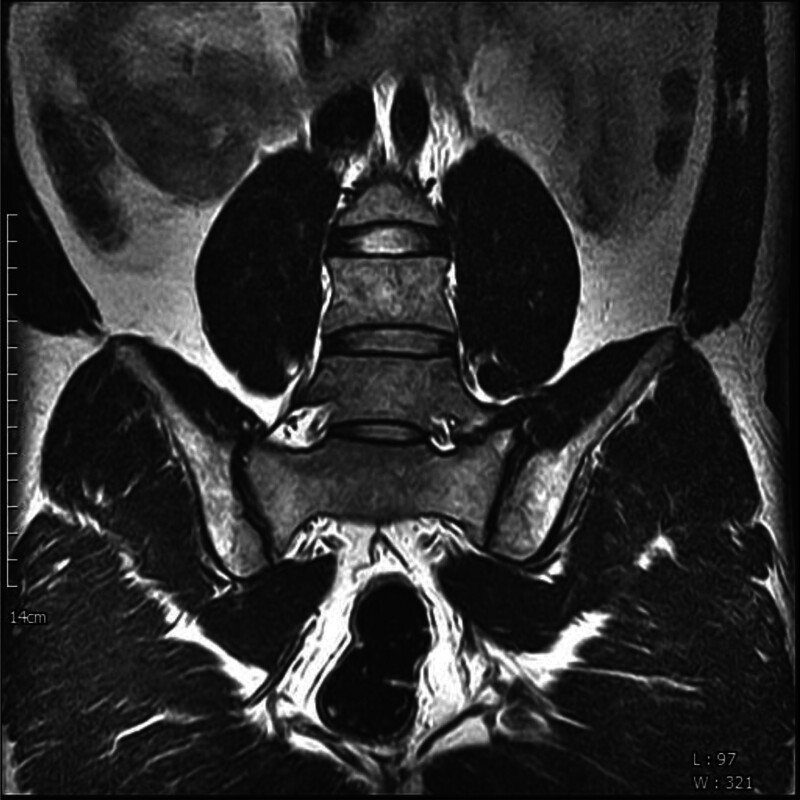
Magnetic resonance image illustrating Bertolotti syndrome.

Bertolotti syndrome (BS) is characterized by low back pain (LBP) and pain in the hip region radiating to the legs, caused by lumbosacral transitional vertebrae (LSTV).^[1–7]^ Diagnosing BS involves confirming LSTV through imaging studies (Figs. 1 and 2) and clinical assessment of symptoms, while ruling out other possible conditions.^[5–9]^ The Castellvi classification system categorizes LSTV into types I to IV based on the connection type between the transverse process and the sacrum.^[10]^ Type I and II LSTVs present with a unilateral or bilateral transverse process, while types III and IV involve partial or complete fusion of LSTVs and the sacrum.^[10]^ Particularly, LSTV of Castellvi type III is associated with LBP; however, further investigation is required to elucidate the causality.^[11]^ LSTV is relatively common with a prevalence of approximately 35% in the total population.^[1,2,5,12–15]^ However, the prevalence of BS is widely debated.^[5,7,8,12,13]^

BS is attributed to a morphological anomaly in the L5/S1 vertebrae, first described by Bertolotti in 1917 as being associated with LBP.^[2,3,5,7,8,16]^ Thereafter, numerous studies with various approaches have been conducted to elucidate the causes of pain in BS. Accelerated degenerative changes in the lumbar spine (L-spine), scoliosis and arthropathy of the joints, and deformation of the transitional vertebral segment causing nerve compression have been reported as possible causes of pain; however, the exact etiology remains controversial.^[2,5,6,8]^ Despite this uncertainty, LSTV often coincides disc herniation and accelerated degenerative changes,^[2–5,8,12]^ highlighting the need for effective BS treatment.

Currently, primary treatments for BS include nonsteroidal anti-inflammatory drugs, local steroidal or anesthetic injections, and physical therapy.^[2,5]^ Although surgical intervention shows a marginally higher efficacy than nonsurgical management,^[2,8]^ both physicians and patients prefer conservative treatment to surgical treatment due to the complex surgical process and possible complications caused by surgery. Thus, there is a need for a safer and more effective treatment alternative.^[3,5,8,12,13,17]^ Prior studies have shown that many patients with musculoskeletal disorders, including those caused by structural changes, frequently use Integrative Korean Medicine (IKM) treatment in Korea for both pain and disability management.^[18,19]^ Therefore, in this study, we report the case of a patient with BS who was treated with IKM, including but not limited to, pharmacopuncture and Chuna manual therapy (CMT), resulting in significant and sustained symptom improvement. Moreover, we aimed to explore the impact of LSTV on LBP by analyzing the clinical features of patients with LSTV according to radiological examinations.

## 2. Case report

### 2.1. Clinical features

A 33-year-old man presented at our hospital with LBP symptoms, numbness/tingling, and dull pain in the left posterior lower extremity (LE) persisting for 4 months. The patient was previously diagnosed with BS at another hospital 1 month earlier. Initially, the pain originated in his lower back and the adjacent pelvic region, but worsened after prolonged periods of sedentary sitting for occupational reasons, resulting in severe radiculopathy from the left thigh to the calf. Therefore, the patient underwent 2 sessions of injection therapy. Despite the injection therapy, the patient’s symptoms worsened. Accordingly, the patient was diagnosed with BS of Castellvi Type II,^[12,16]^ in which the left L5 transverse process formed unilateral pseudo-articulation with the sacral ala. The patient was advised to undergo transverse processectomy.^[16]^ However, due to a reluctance toward surgery, the patient visited the Korean medicine hospital to receive IKM treatment.

At the initial visit, the patient complained of dull pain radiating from the bilateral lower lumbar region to the left posterior LE, along with a tingling sensation around the left groin. With no significant medical or family history and normal laboratory test results, the patient exhibited impaired physical functions in the lumbar region, extreme pain while changing posture, and sleep disturbances due to nocturnal pain. Due to the sense of enervation in the left LE, the patient experienced frequent falls, negatively affecting his daily life. Pain severity was assessed using the Numeric Rating Scale (NRS) and the patient’s condition was assessed using the Euroqol 5-dimension (EQ-5D) index and Oswestry Disability Index (ODI). On admission, the patient’s NRS scores were 5 and 3 points for LBP and radiculopathy, respectively, EQ-5D was 0.437, and ODI was 57.78. On physical examination, the lumbar range of motion could not be appropriately assessed due to severe pain, and the results of the straight leg raise (SLR) test were 45/30°, exhibiting impaired mobility of the bilateral lumbar and pelvic regions. The examination also revealed reduced strength in the muscles innervated by the L4 and L5 nerve roots, as evidenced by big toe extension G4/G3 and ankle dorsiflexion G4/G4. However, there was no significant strength impairment in the muscles innervated by the S1 nerve root, indicated by ankle plantar flexion G5/G5. Moreover, there were no symptoms of hypesthesia. All deep tendon reflex test results were normal, and it was clinically judged that there was no urgent need for surgery. Therefore, the decision was made to manage his symptoms with IKM therapy, including herbal medicine, pharmacopuncture, and CMT.

### 2.2. Treatment

The patient’s interventions are detailed in Table 1. The primary treatments included Korean traditional herbal formulas, specifically Cheongpajeon (CPJ) and Cheongwoongbaro-hwan, which contain herbs such as 7.5 g/d of *Eucommia ulmoides* and 3.75 g/d of *Ostericum koreanum*, both known for their effectiveness in treating musculoskeletal disorders. The herbal formulas, as described in Table 1, were decocted together and divided into 3 doses per day. The patient was instructed to take 1 dose 30 minutes after each meal. In cases where the patient skipped a meal, they were advised to still take the medication at the same approximate time. In addition, variants of CPJ and Cheongwoongbaro-hwan, namely Cheongpayanggeuntang (CPYG) and Yukgongbaro-hwan (YGBR), were administered and prepared with adjustments according to the patient’s clinical condition. The patient received no other medication apart from herbal medicine.

**Table 1:** Details of intervention.

Treatment type	Ingredients	Treatment details and frequency
*Herbal medicine*		
Cheongpajeon (CPJ)	*Eucommia ulmoides* 7.5 g *Acanthopanax sessiliflorus* 7.5 g *Achyranthes bidentata* 7.5 g *Saposhnikovia divaricata*, 7.5 g *Cibotium barometz* 7.5 g *Lycium chinense* 7.5 g, *Boschniakia rossica* 7.5 g *Cuscuta chinensis* 7.5 g Glycine max 7.5 g *Ostericum koreanum* 3.75 g *Atractylodes japonica* 3.75 g *Psoralea corylifolia* 3.75 g	30 minutes after each meal 3 times a day
Cheongwoongbaro-hwan (CWBR) (pills)	*Acanthopanax sessiliflorus* 7.5 g *Cibot rhizome* 2 g *Eleutherococcus sessiliflorus* 7.5 g	
Cheongpayanggeuntang (CPYG)	*Achyranthes bidentata* 7.5 g *Testudinis Plastrum* 2 g *Cibot rhizome* 2 g	
Yukgongbaro-hwan (YGBR) (pills)	*Acanthopanax sessiliflorus* 7.5 g *Velvet Antler* 4 g *Cibot rhizome* 2 g *Eleutherococcus sessiliflorus* 2 g	
*Pharmacopuncture*		
Shinbaro (Jaseng Korean Medical Hospital, Namyangju, Republic of Korea)	*Cibotium barometz,* *Saposhnikovia divaricata,* *Eucommia ulmoides,* *Acanthopanax sessiliflorus,* *Ostericum koreanum,* *Angelica pubescens,* *Achyranthes japonica,* *Paeonia albiflora,* *Scolopendra subspinipes*	2 times a day Disposable syringe (Kovax-Syringe 2 mL, 26 G × 1 1/2)
*Chuna manual therapy* (*CMT*)		
	Side lying position lumbar distraction technique, prone position sacral dysfunction correction technique, prone position iliac dysfunction correction technique	Once a day

Pharmacopuncture aimed to stimulate the left L5 nerve root and adjacent tissues. Therefore, acupoints in the lower lumbar region and sacroiliac joint, including GB30, BL24, BL25, and BL31, were targeted. Each acupoint received an injection of 2 mL of Shinbaro pharmacopuncture, the preparation of which is described in detail elsewhere.^[20]^ Manual acupuncture was also administered at the same acupoints using stainless steel disposable needles for 15 minutes, combined with low-frequency electrical stimulation. Pharmacopuncture and acupuncture treatments were conducted twice daily during inpatient care and once per visit during outpatient care. To address vertebral alignment imbalance, CMT was administered once daily during inpatient care and once per visit during outpatient care by a Korean medical doctor. A variety of techniques were selected based on clinical judgment and the patient’s symptoms, including the side-lying lumbar distraction technique, prone sacral dysfunction correction technique, and prone iliac dysfunction correction technique. Each session lasted 10 to 15 minutes.

**Figure 3.**
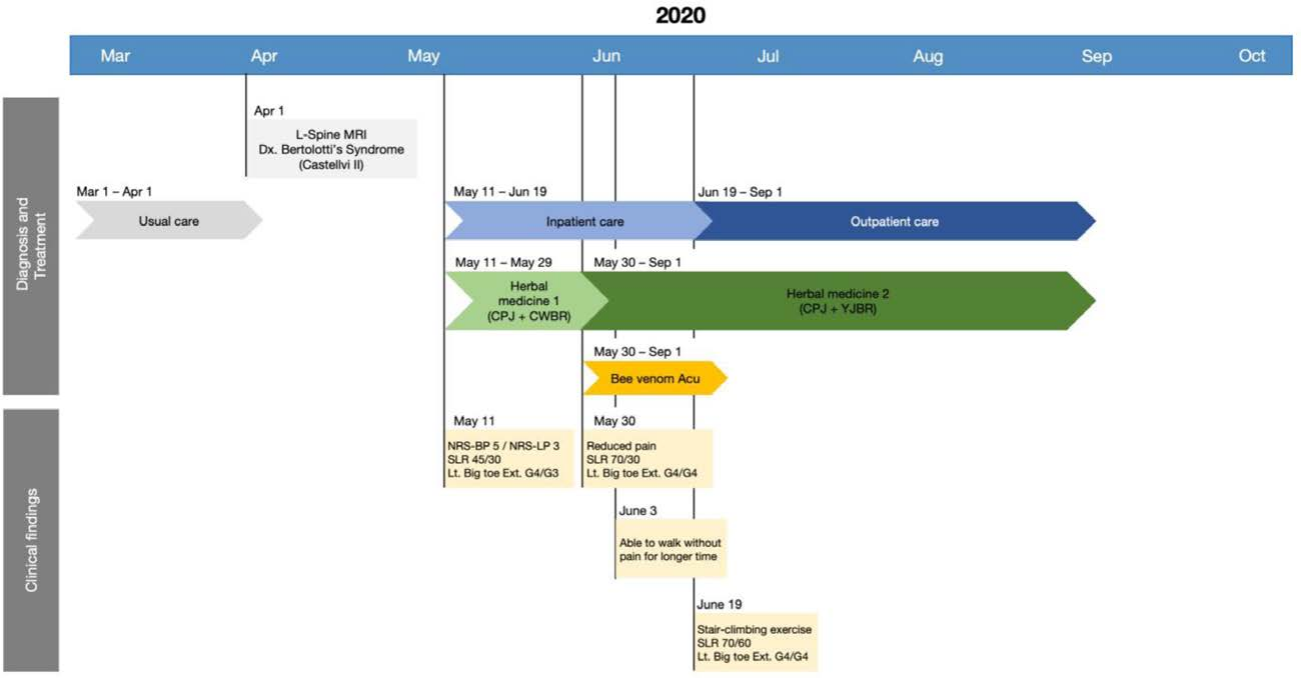
Timeline of the patient’s treatment course. BS = Bertolotti syndrome; CPJ = Cheongpajeon; CPYG = Cheongpayanggeuntang; CWBR = Cheongwoongbaro-hwan; Ext = extension; L-Spine MRI = lumbar-spine magnetic resonance imaging; Lt = left; NRS = numeric rating scale; SLR = straight leg raise; Tx = treatment; YGBR = Yukgongbaro-hwan.

The inpatient IKM treatment regimen continued for 40 days, complemented by standard care, including physical therapy. The patient’s treatment timeline is presented in Figure 3.

### 2.3. Clinical outcomes

**Figure 4.**
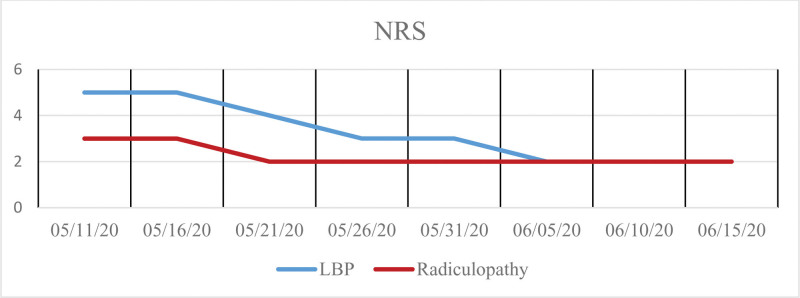
Trend in Numeric Rating Scale scores for low back pack pain and radiculopathy. LBP = low back pain; NRS = Numeric Rating Scale.

Most inpatients receive 2 treatments a day at our facility during inpatient care; the patient underwent treatment 61 times over 40 hospitalization days from May 11 to June 19, with no treatment-free days. The changes in the patient’s symptoms were evaluated on the day of admission, 2 weeks after admission, and at discharge, using the NRS, EQ-5D, and ODI, all of which are patient-reported outcomes. The scores showed gradual improvement at Week 1, Week 2, and 1 month of hospitalization. At discharge, the NRS scores for both LBP and radiculopathy were 2, representing a nearly 50% decrease (Fig. 4), with EQ-5D and ODI scores of 0.70 and 40.00, respectively.

Physical examinations were conducted to provide objective outcome measures on the day of admission and discharge to confirm the diagnosis. The results for outcome measures of physical functions were SLR 45/30, big toe extension G4/G3, ankle dorsiflexion G4/G4, and ankle plantar flexion G5/G5 at admission, suggesting impaired functions in the left lower lumbar and pelvic regions compared to those on the right side. At discharge, the results were SLR 70/60, big toe extension G4/G4, ankle dorsiflexion G4/G4, and ankle plantar flexion G5/G5, indicative of restored muscle strength on the left side and increased mobility in bilateral lumbar and pelvic regions compared to the corresponding at admission.

No adverse events, aside from common acupuncture-related effects, such as subcutaneous hematoma, bleeding, skin bruising, and needle site pain, were reported.^[21]^ The patient sometimes complained of fatigue; however, it was possibly unrelated to the treatment, and may have been caused by long-term hospitalization.

## 3. Multicenter retrospective chart review

To analyze the clinical epidemiological characteristics of patients with LSTV, we conducted a review of medical charts. Patients were included if they had national health insurance (NHI) coverage and radiological findings of LSTV, confirmed through spine radiographs, L-spine computed tomography scans, L-spine magnetic resonance imaging scans, or cervical–thoracic–L-spine magnetic resonance imaging scans. Data were collected from 4 different Korean medicine hospitals from January 1, 2018, to December 31, 2022. The study was approved by the Institutional Review Board of Jaseng Korean Medicine Hospital (Jaseng 2023-02-004). The patient provided informed consent for the case report, including the publication of his radiologic images.

### 3.1. Inclusion and exclusion criteria

**Figure 5.**
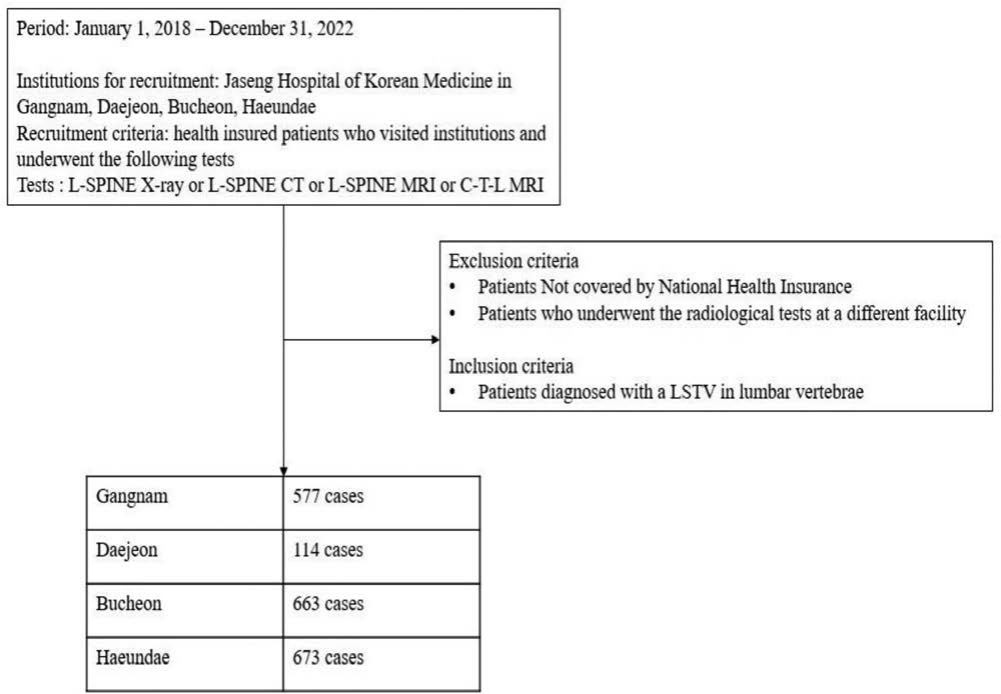
Flowchart of the multicenter clinical epidemiology study. CT = computed tomography; C–T–L = cervical–thoracic–lumbar; LSTV = lumbosacral transitional vertebrae; L-spine = lumbar spine; MRI = magnetic resonance imaging.

The inclusion criteria included patients with NHI coverage, and those with radiological images to confirm the diagnosis. Patients with confirmed diagnoses of “lumbarization,” “sacralization,” or “LSTV” based on radiologist reports were included. The exclusion criteria were those without NHI coverage, those lacking radiological images, and patients whose transitional vertebrae were confirmed in regions other than the L-spine (Fig. 5).

### 3.2. Data extraction

Medical records of patients who met both the inclusion and exclusion criteria were collected from 4 hospitals. Data extraction followed a standardized process across all 4 hospitals, as they shared the same electronic medical record format and data warehouse. The collected data included age, sex, type of LSTV, pain levels, surgical history, surgical segment, and comorbidity status (such as spondylolisthesis and scoliosis) for all eligible patients.

### 3.3. Statistical methods

Descriptive statistical analysis was performed in this study. For the demographic characteristics of LSTV patients, categorical variables were presented using frequency (N) and percentage (%), and continuous variables were presented using the mean and standard deviation (SD). For a detailed understanding of the status and trends of LSTV, in-depth analyses were performed according to sex by lumbarization and sacralization and medical institution type. The statistical suite, SAS V9.4 (SAS Institute Inc, Cary, NC), was used for the calculation of results and tabulation.

## 4. Results

The demographic characteristics of the patients analyzed in this study are shown in Table 2. Most patients with LSTV were in their 40s and 50s, with no significant difference in incidence according to sex. Regarding LSTV type, sacralization was more prevalent in men, reducing the flexibility of vertebrae. Conversely, lumbarization was more frequent in women, increasing the flexibility of vertebrae (Table 3).

**Table 2:** Characteristics of the patients with LSTV from 2018 to 2022.

	n	%
*Age group* (*years*)		
10–19	21	1%
20–29	197	10%
30–39	392	19%
40–49	415	20%
50–59	400	20%
60–69	361	18%
70–79	182	9%
80–89	52	3%
≥90	7	0%
*Sex*		
Male	976	51.9%
Female	1051	48.1%
*Type of pain*		
No pain related to lumbar spine	226	11%
LBP	949	47%
Radiculopathy	12	1%
LBP with radiculopathy	840	41%
*History of surgery*		
Yes	72	4%
No	1955	96%
*Surgical segment*		
L1/2	4	7%
L2/3	5	9%
L3/4	6	11%
L4/5	20	35%
L5/1	22	39%
*Comorbidities*		
*Spondylolisthesis*	292	14%
*Scoliosis*	122	6%

LBP = low back pain, LSTV = lumbosacral transitional vertebrae.

**Table 3:** Correlation of LSTV type and sex.

Type of LSTV		
	Lumbarization	Sacralization
Male	448 (42%)	611 (58%)
Female	586 (57%)	467 (43%)

LSTV = lumbosacral transitional vertebrae.

Pain attributed to LSTV primarily affected the L-spine, although a few patients reported pain in other regions. Despite the malalignment caused by LSTV, there were only a few patients who received surgery (4%) or had other deformities, such as spondylolisthesis (14%) or scoliosis (6%). The patients who received surgery commonly had complications at L5/S1. The type of treatment that the patients received is reported in Table 4. Most of the patients with LSTV underwent outpatient treatment. Indeed, the number of patients who received outpatient treatment was more than double that of patients who received inpatient treatment.

**Table 4:** Treatment type for patients with LSTV.

Treatment type		
Inpatient	547	27%
Outpatient	1480	73%

LSTV = lumbosacral transitional vertebrae.

## 5. Discussion

This study presents a novel case of BS showing improvement with IKM treatment, suggesting a potential alternative management option for BS. The structural deformity associated with BS, specifically the fusion of L5 and S1 vertebrae, often leads to severe radiculopathy and LBP. Our patient, initially scoring 5 on the NRS for pain, experienced worsening symptoms despite injection therapy, leading to a diagnosis of BS and a recommendation for surgery. However, after approximately 40 days of inpatient IKM treatment, the patient’s lumbar-hip range of motion nearly normalized, and LE pain was reduced to an NRS of 2. Continued herbal medicine treatment for 3 months further sustained the improved symptoms and enhanced muscle strength, with a follow-up in October 2020 confirming no limitations in daily life and absence of pain.

Comparatively, previous studies on steroid injections reported a 50% pain reduction after 1 month,^[22]^ while surgical resection of LSTV often left patients with persistent sciatic pain.^[23,24]^ In this context, the improvement observed in our case appears relatively effective and safe.^[25]^ This finding is particularly relevant given that many patients with BS do not respond to steroid injections, necessitating alternative treatment options.

The herbal formula CPJ, containing Eucommia ulmoides and Acanthopanax sessiliflorus, has been widely used for musculoskeletal disorders, with documented efficacy in several cases. Acupoints such as GB30, BL24, BL25, and BL31 are also commonly used for lumbar and pelvic symptoms.^[26–28]^ Additionally, CMT, which aims to enhance function by aligning the L-spine and soft tissues, has shown effectiveness in previous studies.^[29–33]^

This study contributes to the literature by reporting the first case of BS managed with IKM intervention, with the patient’s improvement measured using the NRS, EQ-5D, and ODI. These results suggest that IKM could be a viable and safe alternative treatment for managing severe cases of BS.

In our epidemiological study, we found that the prevalence of LSTV causing BS is approximately 30%, consistent with prior research.^[2,5,8]^ The incidence was generally higher in men, with sacralization more common in men and lumbarization more prevalent in women.^[5,8]^ Our findings also revealed low rates of scoliosis (6%) and spondylolisthesis (14%) among patients with LSTV, which contrasts with higher rates reported in the literature.^[6,34]^ This discrepancy may be attributed to variations in how physicians recorded the patients’ medical histories.

Among patients with LSTV, only 3.6% had a history of surgery, with the L5/S1 segment being the most frequently reported surgical site, followed by the L4/L5 segment. This aligns with previous findings that disc herniation often occurs in the segment immediately superior to the LSTV lesion.^[2,4–6,8,35,36]^ To explore the correlation between LSTV and LBP, we calculated the proportion of patients with confirmed LSTV on radiological examinations but without LBP/radiculopathy. We found that 11% of patients with confirmed LSTV visited the hospital for reasons other than lumbar symptoms, which aligns with a previous study reporting that 13% of patients with confirmed LSTV were asymptomatic.^[14,16,37]^ These results suggest that not all LSTV cases cause pain. Additionally, 73% of patients with LSTV received outpatient treatment, with only 27% requiring inpatient care, indicating that most patients experience mild to moderate disability.

This study had several limitations. First, as BS is relatively rare, and no other cases were reported to our institution during the study period, establishing a control group or conducting a randomized controlled trial (RCT) was not feasible. Consequently, our findings, while valuable, are insufficient to be presented as established evidence for the effectiveness of IKM treatment for BS. Moreover, owing to the nature of IKM treatment, which combines multiple modalities such as acupuncture, pharmacopuncture, electroacupuncture, moxibustion, cupping, and CMT, it was challenging to determine the effect of each individual treatment mode. The epidemiological analysis was conducted solely among patients who visited the 4 hospitals for treatment, resulting in a small sample size that may not be representative of the general population. Furthermore, the radiological readings were conducted separately at each hospital, which may have introduced a potential lack of consistency between the reports. Additionally, the retrospective review of medical records may have introduced potential inconsistencies and inaccuracies in the information analyzed. To strengthen the evidence base, future studies should include a comparison group receiving standard care or placebo treatments. Furthermore, additional research on IKM treatment for patients with BS, as well as broader epidemiological analyses based on publicly released data, is necessary to better understand and validate these findings.

In conclusion, this case study provides preliminary evidence supporting the effectiveness of IKM treatment for managing BS. Although our findings offer valuable insights, further research is needed to corroborate and validate these results, particularly given the lack of existing studies on IKM’s effects on BS. This study also reports the epidemiological characteristics of patients with LSTV, despite limitations such as the small sample size and the inclusion of patients from only 4 specific hospitals. Future research could involve large-scale follow-up studies to build upon our findings and provide a more comprehensive understanding of BS and its management.

## Author contributions

**Conceptualization:** Sohyun Cho, Yong-Jun Ahn.

**Data curation:** Sohyun Cho.

**Formal analysis:** Sohyun Cho, Yoon Jae Lee, Ye-Seul Lee.

**Investigation:** Sohyun Cho, Yong-Jun Ahn.

**Supervision:** Ye-Seul Lee.

**Writing – original draft:** Sohyun Cho.

**Writing – review & editing:** In-Hyuk Ha, Ye-Seul Lee.
